# Wheat PP2C-a10 regulates seed germination and drought tolerance in transgenic Arabidopsis

**DOI:** 10.1007/s00299-020-02520-4

**Published:** 2020-02-17

**Authors:** Xiaofen Yu, Jiapeng Han, Li Li, Qian Zhang, Guangxiao Yang, Guangyuan He

**Affiliations:** The Genetic Engineering International Cooperation Base of Chinese Ministry of Science and Technology, Key Laboratory of Molecular Biophysics of Chinese Ministry of Education, College of Life Science and Technology, Huazhong University of Science and Technology, Wuhan, China

**Keywords:** Wheat, Seed germination, DOG1, PP2C, ABA

## Abstract

**Key message:**

A wheat protein phosphatase PP2C-a10, which interacted with TaDOG1L1 and TaDOG1L4, promoted seed germination and decreased drought tolerance of transgenic Arabidopsis.

**Abstract:**

Seed dormancy and germination are critical to plant fitness. *DELAY OF GERMINATION 1* (*DOG1*) is a quantitative trait locus for dormancy in *Arabidopsis thaliana*. Some interactions between DOG1 and the type 2C protein phosphatases (PP2Cs) have been reported in Arabidopsis. However, the research on molecular functions and regulations of DOG1Ls and group A PP2Cs in wheat (*Triticum aestivum*. L), an important crop plant, is rare. In this study, the whole *TaDOG1L* family was identified. Expression analysis revealed that *TaDOG1L2*, *TaDOG1L4* and *TaDOG1L-N2* specially expressed in wheat grains, while others displayed distinct expression patterns. Yeast two-hybrid analysis of TaDOG1Ls and group A TaPP2Cs revealed interaction patterns differed from those in Arabidopsis, and TaDOG1L1 and TaDOG1L4 interacted with TaPP2C-a10. The qRT-PCR analysis showed that *TaPP2C-a10* exhibited the highest transcript level in wheat grains. Further investigation showed that ectopic expression of *TaPP2C-a10* in Arabidopsis promoted seed germination and decreased sensitivity to ABA during germination stage. Additionally, *TaPP2C-a10* transgenic Arabidopsis exhibited decreased tolerance to drought stress. Finally, the phylogenetic analysis indicated that *TaPP2C-a10* gene was conserved in angiosperm during evolutionary process. Overall, our results reveal the role of *TaPP2C-a10* in seed germination and abiotic stress response, as well as the functional diversity of *TaDOG1L* family.

**Electronic supplementary material:**

The online version of this article (10.1007/s00299-020-02520-4) contains supplementary material, which is available to authorized users.

## Introduction

Seed dormancy is critical to plants by preventing germination under unfavorable conditions. Insufficient dormancy can lead to pre-harvest sprouting which significantly decreases the grain yield and quality in agricultural production, however too deep dormancy delays germination, thus decreasing the growth time at the appropriate season (Gubler et al. 2005; Finch-Savage and Leubner 2006).

The induction and release of dormancy are mainly controlled by two plant hormones: abscisic acid (ABA) and gibberellins (GA) (Yan and Chen 2017; Vishal and Kumar 2018). ABA participates in the processes of plant growth, development and responses to various abiotic stresses, while GA is involved in various stages of plant growth and development. ABA and GA have antagonistic effects on seed dormancy and germination, i.e., endogenous ABA content is gradually decreased from dormancy to germination phase in the seed, whereas the GA level is increased during this process (Golldack et al. 2013; Shu et al. 2016). In Arabidopsis, ABA-deficient mutants accelerate germination (Frey et al. 2011), while ABA catabolism mutants display deeper dormancy phenotype (Matakiadis et al. 2009).

In addition to ABA synthesis- and catabolism-related factors, proteins in ABA signaling pathway also affect seed dormancy and germination. ABA insensitive 3 (ABI3), ABI4, and ABI5 which are the transcriptional regulators of ABA signaling, are involved in seed dormancy and germination in Arabidopsis (Söderman et al. 2000; Finkelstein et al. 2002). MOTHER OF FT AND TFL1 (MFT), a phosphatidyl ethanolamine-binding protein (PEBP), plays a role in both ABA and GA signaling pathways to regulate seed germination in Arabidopsis (Xi et al. 2010). Group A protein phosphatase 2Cs (PP2Cs) which are ABA co-receptors, negatively regulate ABA signaling pathway. ABI1 and ABI2 are the group A PP2C proteins in Arabidopsis, their single mutants exhibited reduced sensitivity to exogenous ABA at seed germination stage (Leung et al. 1997; Gosti et al. 1999). However, their double mutant displays increased response to ABA comparing to the single mutants, suggesting that ABI1 and ABI2 form a negative feedback loop in regulating ABA signaling pathway (Merlot et al. 2001). Another two group A PP2Cs in Arabidopsis, ABA hypersensitive germination 1 (AHG1) and AHG3/AtPP2CA, play distinct and overlapping roles in seed germination (Yoshida et al. 2006; Nishimura et al. 2007). *HON*, a group A *PP2C* specifically expresses in seeds, inhibits ABA signaling directly while activating GA signaling indirectly in imbibed seeds, thus regulating seed dormancy homeostatically (Kim et al. 2013). The reduced dormancy 5 (RDO5), an ungrouped PP2C, suppresses *Arabidopsis PUMILIO 9* (*APUM9*) transcript levels to regulate seed dormancy (Xiang et al. 2014).

Apart from phytohormone related proteins, other key regulators of seed dormancy have been characterized. *DELAY OF GERMINATION 1* (*DOG1*) is identified as quantitative trait locus for dormancy in Arabidopsis (Alonso-Blanco et al. 2003). *DOG1* belongs to a novel gene family which is plant-specific; other four members *DOG1-like 1*–*4* (*DOGL1*–*4*) have been identified. *DOG1* specifically expresses in the seed, and displays sequence diversity in the coding regions of different Arabidopsis varieties (Bentsink et al. 2006). DOG1 can regulate seed dormancy in an ABA-independent way (Nakabayashi et al. 2012). Further study has demonstrated that DOG1 acts as a timer for seed germination in a temperature-dependent manner (Graeber et al. 2014). Besides, DOG1 also affects seed development via genetic interaction with ABI3 (Dekkers et al. 2016), flowering time via microRNA pathway (Huo et al. 2016). It was assumed that DOG1 functioned in parallel to ABA signaling as both ABA and DOG1 have effects on seed dormancy (Nakabayashi et al. 2012). Recent studies found that DOG1 directly interacted with AHG1 and AHG3, which were members of group A PP2Cs, to control seed dormancy (Née et al. 2017; Nishimura et al. 2018).

Seed dormancy is an important agronomic trait for cereals, especially for wheat (*T. aestivum*. L) which is the most widely cultivated cereal around the world. Wheat dormancy and pre-harvest sprouting can be affected by TaDOG1Ls. Four *DOG1-like* genes in wheat have been identified, including *TaDOG1L1*, *TaDOG1L2*, *TaDOG1L4* and *TaDOG1L5-1* (Ashikawa et al. 2013). Ectopic expression of *TaDOG1L1* increased seed dormancy in Arabidopsis (Ashikawa et al. 2010). Overexpression and RNA interference of *TaDOG1L4* in wheat confirmed the role of *TaDOG1L4* in seed dormancy and germination (Ashikawa et al. 2014). The fully annotated reference genome of hexaploid wheat has been completed; this greatly facilitates the identification of gene families in wheat genome. In this study, genome-wide identification and expression analyses of *TaDOG1L* genes were conducted. Several group A *TaPP2C*s were isolated in our previous study (Yu et al. 2019). To examine the regulation of TaDOG1Ls by group A TaPP2Cs in wheat, yeast two-hybrid and bimolecular fluorescence complementation (BiFC) assays were performed and TaPP2C-a10 was found to interact with TaDOG1L4 in nuclei. Subsequently, the role of *TaPP2C-a10* and possible regulatory mechanism in seed dormancy and germination were investigated in Arabidopsis. In addition to seed dormancy, TaPP2C-a10 also functioned in drought stress response. These findings enrich the functional studies of TaDOG1Ls and group A PP2Cs in wheat.

## Materials and methods

### Identification and phylogenetic analysis of TaDOG1L in wheat genome

To identity all the TaDOG1L proteins, DOG1 domain (PF14144) in Pfam 32.0 database (https://pfam.xfam.org) (El-Gebali et al. 2018) was used to search against the wheat annotated reference genome database (IWGSC RefSeq v1.0) by the hmmsearch program of the HMMER software 3.2.1 (https://hmmer.org/download.html) (Wheeler and Eddy 2013). The obtained proteins were further screened for conserved domains by InterProScan (https://www.ebi.ac.uk/interpro/download/) (Mitchell et al. 2018) in STANDALONE mode and NCBI CD-Search tool (https://www.ncbi.nlm.nih.gov/Structure/cdd/wrpsb.cgi) (Marchler-Bauer et al. 2014). Protein sequences containing DOG1 domains were used to perform multiple sequence alignment by ClustalX 2.1 (Larkin et al. 2007), and then the phylogenetic tree was constructed by MEGA 6.0 based on the neighbor-joining (NJ) method with 1000 bootstrap replicates (Tamura et al. 2011). To analyze the exon–intron structures of *TaDOG1L*s, the coding sequences and genome sequences were downloaded from Ensembl Plants database (https://plants.ensembl.org/index.html) (Howe et al. 2019). Gene Structure Display Server was then used to determine and visualize gene structures (https://gsds.cbi.pku.edu.ch) (Hu et al. 2014). To identify the AHG-like proteins in various species, amino sequences of Arabidopsis AHG1 and AHG3 were used to BLASTp against the annotated reference genome databases of *A. thaliana* (TAIR10), *Brassica napus* (AST_PRJEB5043_v1), *Glycine max* (Glycine_max_v2.1), *Medicago truncatula* (MedtrA17_4.0), *Capsicum annuum* (ASM51225v2), *Nicotiana attenuata* (NIATTr2), *Brachypodium distachyon* (Brachypodium_distachyon_v3.0), *Oryza sativa Japonica Group* (IRGSP-1.0), *Aegilops tauschii* (Aet_v4.0), *Hordeum vulgare *subsp.* Vulgare* (IBSC_v2), *T. aestivum*, *Sorghum bicolor* (Sorghum_bicolor_NCBIv3), *Zea mays* (B73_RefGen_v4), *Amborella trichopoda* (AMTR1.0), *Selaginella moellendorffii* (v1.0), *Physcomitrella patens* (Phypa_V3). Proteins with highest score and similarity were selected. Multiple sequence alignment was done by ClustalX 2.1, poorly aligned regions were removed, and then the polygenic tree was constructed by IQ-TREE web server (https://iqtree.cibiv.univie.ac.at/) (Trifinopoulos et al. 2016) using maximum-likehood (ML) method with 1000 bootstrap replicates. Subsequently, the phylogenetic trees were annotated and colored by Evolview webserver (https://www.evolgenius.info/evolview/) (Subramanian et al. 2019). Conserved motifs were discovered by MEME tool (https://meme-suite.org/tools/meme) (Bailey et al. 2006). Approximately 2 kb upstream genome sequence of gene was obtained to analyze the *cis*-regulatory elements by PlantCARE search tool (https://bioinformatics.psb.ugent.be/webtools/plantcare/html/) (Lescot et al. 2002).

### Expression analysis of *TaDOG1L* family in silico

To perform the expression analysis of *TaDOG1L* genes in different tissues at different wheat developmental stages, pubic RNA-seq data of the project choulet_URGI (Ramirez-Gonzalez et al. 2018) and ERP004505 (Pfeifer et al. 2014) were downloaded from the Wheat Expression Browser powered by expVIP (https://www.wheat-expression.com/) (Borrill et al. 2016). The transcripts per million (TPM) values were log 2 transformed to create heatmap by pheatmap package of R project (https://www.r-project.org/).

### The plasmid construction of *TaDOG1Ls* and *TaPP2C-a10*

Primers were designed to isolate *TaDOG1L* genes from mixed cDNA templates of wheat by PCR, and A homoeolog of *TaDOG1L1*, B homoeolog of *TaDOG1L2* and A homoeolog of *TaDOG1L4* were cloned. For yeast two-hybrid assay, the coding regions of *TaDOG1L1*, *2* and *4* were amplified and inserted into vector pGBKT7 via DNA recombination. The pGADT7-*TaPP2C*s plasmids were obtained from our lab (Yu et al. 2019). For subcellular localization assay, the coding sequences of *TaDOG1L1*, *4* and *TaPP2C-a10* were cloned into vector pBI121 containing *eGFP* reporter gene. For BiFC assay, the coding sequences of *TaDOG1L*s and *TaPP2C-a10* were cloned and inserted into SpYCE and SpYNE, respectively. For Arabidopsis transformation, the coding region of *TaPP2C-a10* was inserted into vector pSN1301 containing *GUS* reporter gene. *TaPP2C-a10* and *GUS* gene were driven by individual CaMV 35S promoter. All the primers used for cloning and plasmid construction are listed in Supplementary Table S1.

### Yeast two-hybrid assay

Yeast two-hybrid assay was performed by the Matchmaker GAL4 system (Clontech, USA). The pGADT7-*TaPP2C*s, pGBKT7-*TaDOG1L*s and the control vectors were co-transformed into yeast strain AH109. Interaction of SV40T and p53 or lamin-C was used as positive or negative control, respectively. The transformants were first selected by growing on double-dropout medium (SD/–Trp–Leu, DDO), then appraised by transferring to triple-dropout medium (SD/–His–Trp–Leu, TDO) and quadruple-dropout medium (SD/–Ade–His–Trp–Leu, QDO).

### Subcellular localization and BiFC assays

Vectors pBI121-*TaDOG1L*s, pBI121-*TaPP2C-a10*, SpYCE-*TaDOG1L*s and SpYNE-*TaPP2C-a10* were transformed into *Agrobacterium tumefaciens* strain EHA105, respectively. The positive transformants were then cultured and injected into young leaves of 4-week-old tobacco (*Nicotiana tabacum*) by Agrobacterium-mediated infiltration. For BiFC assay, leaves were co-infiltrated with mixtures of an equal amount of SpYCE/SpYNE culture. After 48 h, GFP or YFP signals of the epidermal cell from the infiltrated leaves were checked by fluorescence microscopy (OLYMPUS LX71, Japan). Cell nuclei were stained by the 4′,6-diamidino-2-phenylindole (DAPI) dye.

### Plant materials and growth conditions

The wheat cultivar Chinese Spring was grown in a greenhouse (16 h light/8 h dark cycle at 22 ℃). Roots, stems, leaves from seedlings at three leaf stage, roots, stems, leaves, flag leaves, pistils, stamens from mature plants at flowering stage, and grains at different days post anthesis (dpa) were collected to perform tissue-specific expression analysis. The Arabidopsis wild-type Columbia was used in this study. Arabidopsis seeds were first sterilized and sown on 1/2 Murashige and Skoog (MS) medium, and then transferred to greenhouse. The whole plants of 7- or 10-day-old seedlings were collected for ABA- or drought-responsive gene expression analysis. All samples were stored at – 80 ℃ before extraction.

### Total RNA extraction and qRT-PCR analysis

Total RNA from tissue sample was isolated by Plant Total RNA extraction Kit (Zomanbio, China), subsequently examined by agarose gel electrophoresis. Then, the first-strand cDNA was created by FastKing RT Kit (Tiangen, China). The qRT-PCR analysis was conducted using the AceQ qPCR SYBR Green Master Mix (Vazyme, China) on a real-time PCR instrument (CFX96; Bio-Rad, USA). The reaction procedure was as follow: 95 ℃ for 5 min, 40 cycles of 95 ℃ for 10 s, 58 ℃ for 20 s, 72 ℃ for 20 s. The 2^−ΔΔ*C*T^ method was used for qRT-PCR analysis (Livak and Schmittgen 2001). The wheat *actin* and Arabidopsis *actin* genes were used as reference genes. All primers used for qRT-PCR are listed in Supplementary Table S1.

### Generation of transgenic Arabidopsis plants

The pSN1301-*TaPP2C-a10* plasmid was transformed into strain EHA105. Positive transformants were cultured to infiltrate Arabidopsis through the floral-dip method (Clough and Bent 1998). Transgenic seeds were selected by growing on 1/2 MS medium (pH 5.8) with 20 mg/L hygromycin B. In addition, GUS staining and PCR detection were used to confirm the transgenic lines. Homozygous lines were used for further analysis. The abundance of the *TaPP2C-a10* transcript in transgenic Arabidopsis was assessed by RT-PCR.

### Germination and root growth assays of Arabidopsis

For germination assay, about 50 seeds were sown on 1/2 MS plates with various concentrations of ABA. Germination (radicles emergence) and post-germination growth (expanded green cotyledons) were counted daily for 7 days. For the root growth assay, 5-day-old seedlings from hormone-free 1/2 MS were transferred to 1/2 MS plates with various concentrations of ABA. The primary root length was measured after 7 days. To analyze the expression levels of ABA-responsive genes in transgenic Arabidopsis and the wild type, 7-day-old seedlings from 1/2 MS plates without ABA were collected for qRT-PCR assay.

### Drought treatment and water loss assay of Arabidopsis plants

For drought treatment, seedlings were grown in soil for 4–5 weeks, then were deprived of water for 7 days before the bolting stage. For water loss assay, rosette leaves were collected from 4-week-old plants, and then immediately weighed at each time point in the lab environment (23–25 ℃). For dry treatment of 10-day-old seedlings, whole plants were exposed in the air of the lab for 1 h. The untreated seedlings were taken as controls to perform qRT-PCR analysis of drought-responsive genes.

## Results

### Genome-wide identification of *TaDOG1L* family

**Fig. 1:**
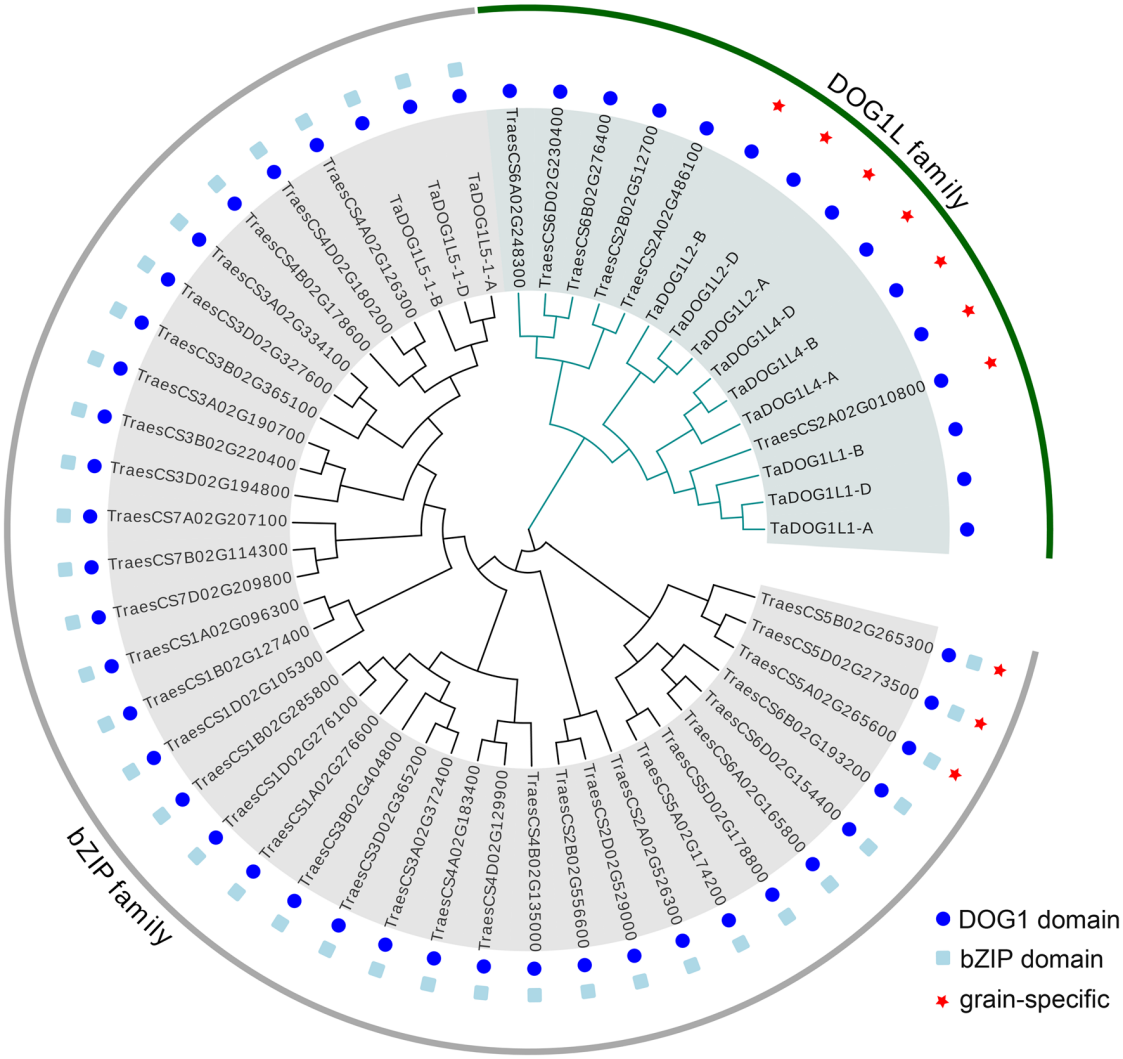
Phylogenetic analysis of all wheat proteins containing DOG domains. Amino acid sequences of 54 wheat proteins were used to construct the phylogenetic tree using the NJ method by ClustalX 2.1 and MEGA 6.0 with 1000 bootstrap replicates. Conserved domains and gene expression pattern are indicated with different shapes

**Fig. 2:**
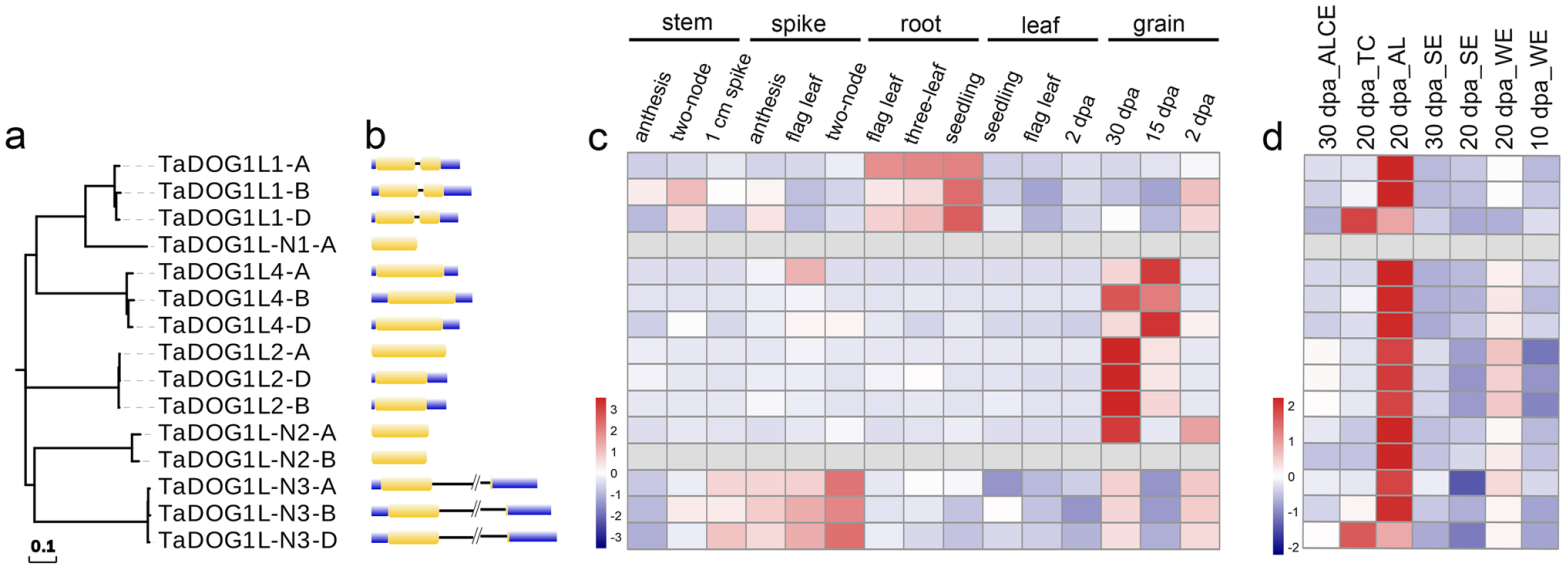
Gene structures and expression patterns of *TaDOG1L* gene family. **a** The phylogenetic tree of *TaDOG1L* family. The scale bar indicates the average number of amino acid substitutions per site. **b** Exon–intron structures of *TaDOG1L* genes. **c** Expression profiles of *TaDOG1L* genes at different developmental stages and tissues. *dpa* days post anthesis. **d** Expression profiles of *TaDOG1L* genes in different cell types at three developmental stages of wheat endosperm. Cell types include whole endosperm (WE), starchy endosperm (SE), aleurone layer (AL), aleurone layer and starchy endosperm (ALCE), transfer cells (TCs). The color scale below represents the relative gene expression level, and red or blue indicates relative higher or lower expression level (**c**, **d**) (color figure online)

Common wheat is allohexaploid with three homoeologous subgenomes (A, B, and D), therefore, each gene in common wheat should potentially have three homoeologs. Previous study has identified four *DOG1-like* genes (12 homoeologs) in wheat based on the amino acid sequence similarity with *AtDOG1* (Ashikawa et al. 2013). In this study, hmmsearch program was applied to search *DOG1L* genes using the latest wheat reference database. After genome-wide searching, 54 proteins were found to have the DOG1 domains (Fig. 1, Table S2). However, there were 39 proteins also containing the typical bZIP domains. Further phylogenetic analysis indicated that these 39 proteins belonged to the clade D bZIP transcription factors including three previous identified proteins of *TaDOG1L5-1* homoeologs. Therefore, a total of six *TaDOG1L* genes (15 homoeologs) were identified including three genes *TaDOG1L1*, *TaDOG1L2* and *TaDOG1L4* which had been identified before (Fig. 1). Another new *TaDOG1L* genes were renamed as *TaDOG1L-N1*, *-N2* and *-N3* (Fig. 2a, Table S2). *TaDOG1L1*, *TaDOG1L2*, *TaDOG1L4* and *TaDOG1L-N3* genes having three homoeologs, *TaDOG1L-N2* lacking D homoeolog, and *TaDOG1L-N1* only having A homoeolog. These genes were located on wheat chromosomes 1, 2, 3 and 6, respectively. In Arabidopsis, except for *DOG1*, four *DOG1-like* genes (*AtDOGL1*-*4*) were identified (Bentsink et al. 2006), so wheat contained one more *DOG1-like* member than Arabidopsis. Unlike *AtDOG1* which had many transcripts (Bentsink et al. 2006; Cyrek et al. 2016), each *TaDOG1L* had only one transcript. Analyses of the exon–intron structures of *TaDOG1L*s revealed that most genes had one exon (Fig. 2b), while *AtDOG1* had three exons.

### Tissue-specific expression analysis of *TaDOG1L* family

Pubic RNA-seq data of wheat variety Chinese Spring were acquired to analyze the tissue-specific expression patterns of *TaDOG1L*s in 15 tissues (stem, spike, root, leaf, grain tissues at three developmental stages) under non-stress condition (Fig. 2c). *TaDOG1L2*, *TaDOG1L4* and *TaDOG1L-N2* exhibited grain-specific expression especially in grains at 15 and 30 dpa stages, excepting that *TaDOG1L4-A* also expressed in spike at flag leaf stage. *TaDOG1L1* had broad range of expression levels in stems, spikes, roots and grains, and preferentially expressed in roots at all developmental stages. *TaDOG1L-N3* displayed non-specific expression in stems, spikes and grains. Overall, all the *TaDOG1L*s expressed in grains except for two homoeologs without detectable expression value. Furthermore, the expression patterns of *TaDOG1L*s in different cell types at three different developmental stages of endosperms were evaluated (Fig. 2d). As a result, most *TaDOG1L*s displayed higher transcript levels in 20 dpa endosperms, mainly in the aleurone layer (AL), merely in starchy endosperm (SE). *TaDOG1L1-A* and *TaDOG1L-N3-A* showed higher transcript abundance in transfer cells (TCs) than AL comparing to their B and D homoeologs, indicating the expression divergence within homoeologs.

### Interactions between TaDOG1Ls and group A TaPP2Cs

**Fig. 3:**
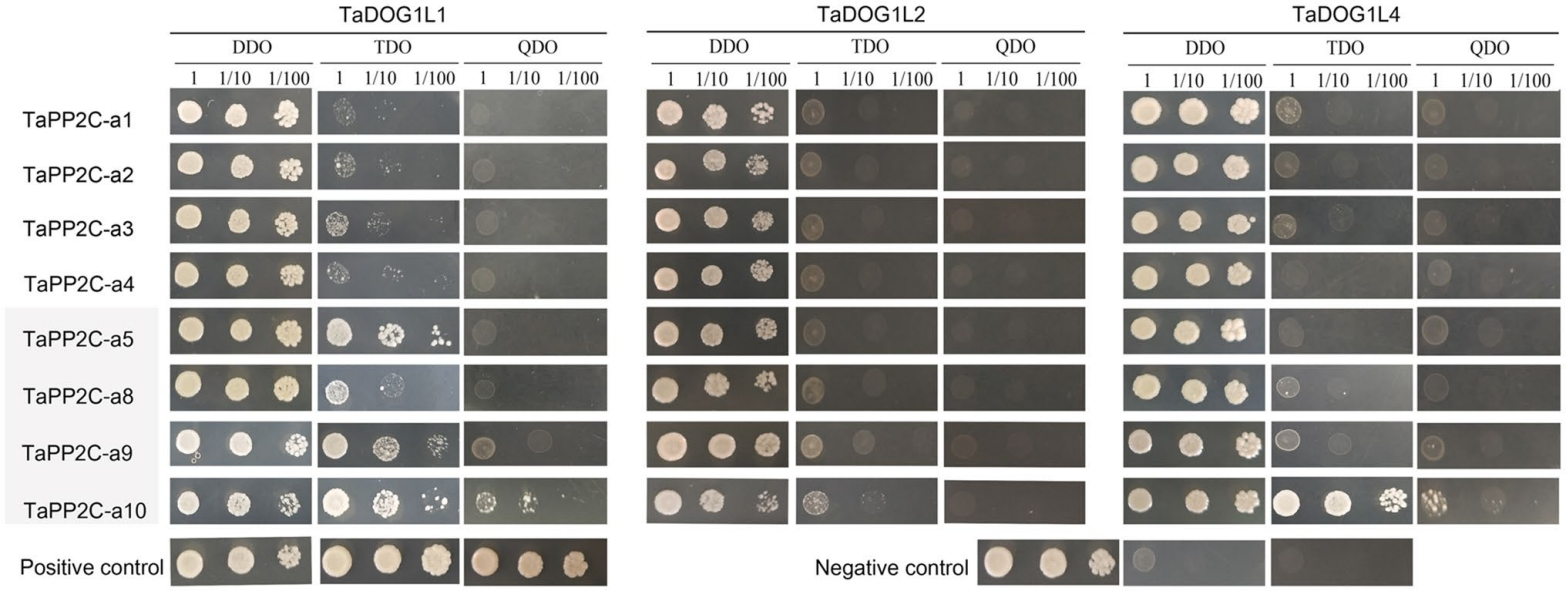
Yeast two-hybrid analysis of TaDOG1Ls with group A TaPP2Cs. Positive transformants were cultured on selective medium DDO (SD/–Leu/–Trp), TDO (SD/–His–Trp–Leu) and QDO (SD/–Ade–His–Trp–Leu), respectively. Yeast strains were assessed at different dilution rates (1, 1/10, and 1/100)

**Fig. 4:**
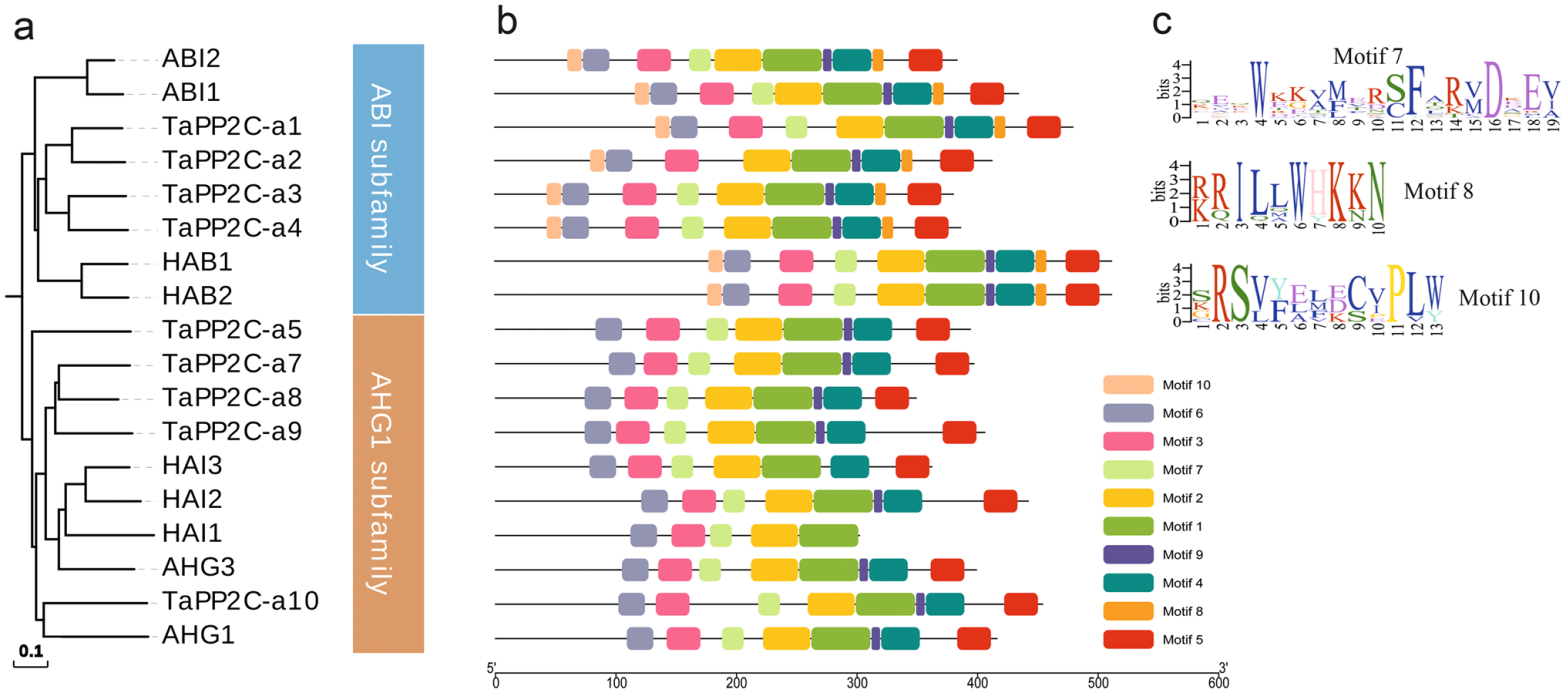
Phylogenetic analysis and motif distribution pattern of group A TaPP2Cs and AtPP2Cs. **a** Phylogenetic analysis of group A TaPP2Cs and AtPP2Cs. The phylogenetic tree was constructed using the NJ method with ClustalX 2.1 and MEGA 6.0 with 1000 bootstrap replicates. The scale bar represents 0.1 amino acid substitutions per site. **b** Motif distribution pattern of group A TaPP2Cs and AtPP2Cs. Ten motifs were discovered by MEME searching tool. The scale bar below corresponds to the length of amino acid sequence. **c** Sequence logos of motif 7, 8 and 10

Three *TaDOG1L*s (*TaDOG1L1*, *2* and *4*) were cloned to perform interaction analysis between TaDOG1Ls and group A TaPP2Cs by yeast two-hybrid assay (Fig. 3). TaDOG1L1 had strong interaction with TaPP2C-a10, and weak interactions with TaPP2C-a5 and -a9. TaDOG1L4 also interacted with TaPP2C-a10. However, no interaction was found between TaDOG1L2 and the eight group A TaPP2Cs. Phylogenetic analysis of all tested TaPP2Cs and group A AtPP2Cs revealed that TaPP2C-a1, -a2, -a3 and -a4 belonged to the ABI subfamily, while TaPP2C-a5, -a8, -a9 and -a10 belonged to the AHG1 subfamily (Fig. 4a). Besides, TaPP2C-a10 was closer to AHG1 than the other three proteins. To discover motifs within group A TaPP2Cs and AtPP2Cs, MEME motif search tool was applied. As a result, ten motifs were identified (Fig. 4b, Table S3). After comparing with the motifs in AtPP2Cs and TaPP2Cs identified by previous studies (Xue et al. 2008; Yu et al. 2019), seven motifs were found to exist in all PP2Cs including group A PP2Cs, and motifs 7, 8 and 10 were specifically found in group A PP2Cs. Moreover, motifs 8 and 10 only existed in ABI subfamily (Fig. 4c).

**Fig. 5:**
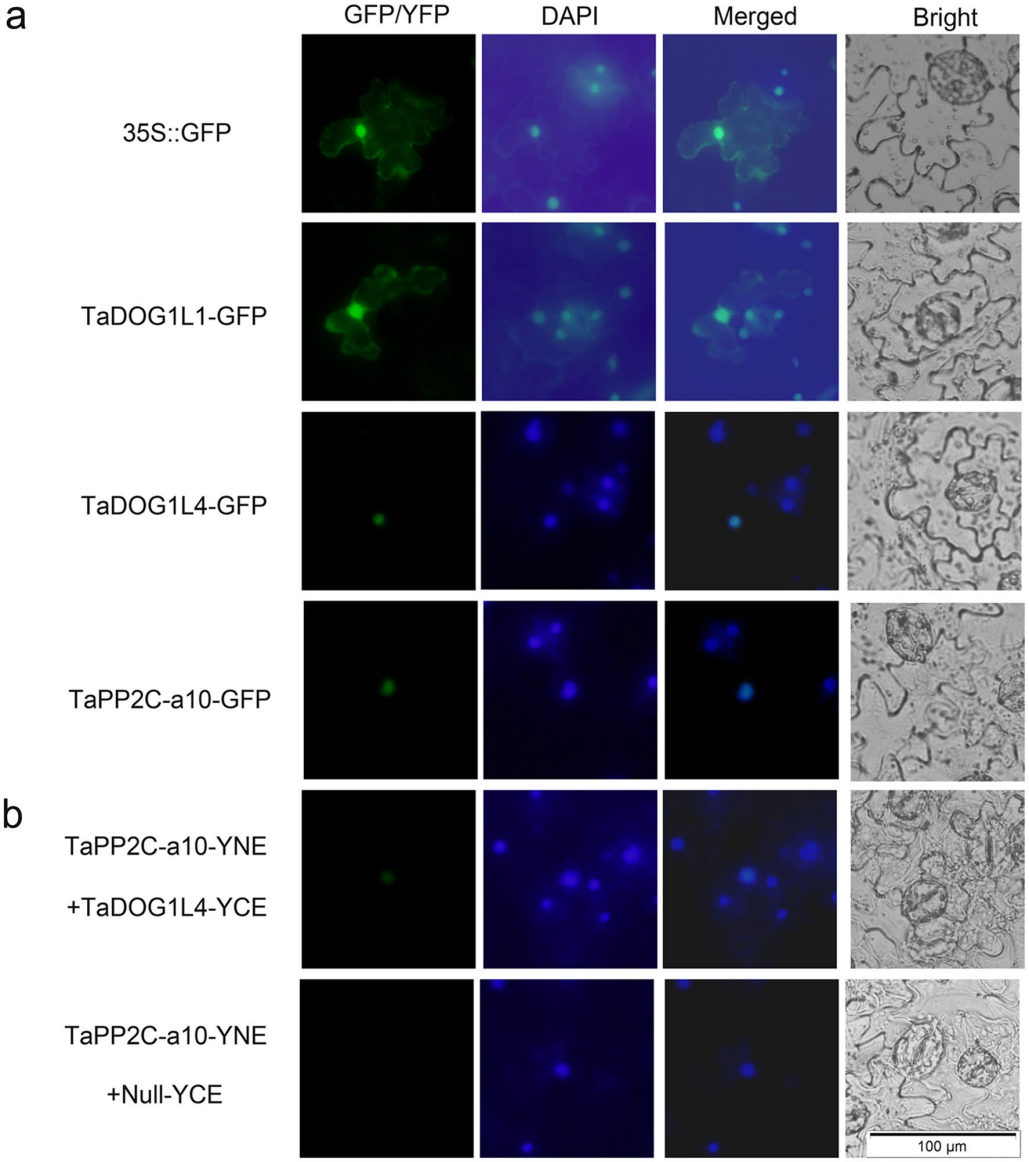
The subcellular localization and interactions of TaDOG1Ls and TaPP2C-a10. **a** The subcellular localization of TaDOG1L1, TaDOG1L4 and TaPP2C-a10 in tobacco leaves. The pBI121-GFP vector was transformed as control. **b** BiFC analysis of TaDOG1L4 and TaPP2C-a10. Leaves were co-infiltrated with plasmids expressing TaPP2C-a10 fused with YNE and DOG1L4 fused with YCE. The GFP and YFP signals were observed after 48–72 h. Scale bars 100 μm

To further confirm the interactions of TaDOG1L1, TaDOG1L4 with TaPP2C-a10 in vivo, BiFC assay was performed using young tobacco leaves. Before BiFC assay, subcellular localizations of TaDOG1L1, TaDOG1L4 and TaPP2C-a10 were determined by transient expressions of their corresponding coding sequences fused with GFP. The locations of nuclei were confirmed by DAPI staining. As shown in Fig. 5a, TaDOG1L1 and TaDOG1L4 exhibited distinct subcellular localization patterns. For TaDOG1L1-GFP fusion protein, green fluorescence signals were detected in both cytoplasm and nuclei, while TaDOG1L4-GFP fusion protein accumulated only in nuclei. Moreover, TaPP2C-a10-GFP fusion protein also displayed nuclear localization. In BiFC assay, fluorescence signals were observed in nuclei when co-expressing *TaPP2C-a10-YNE* and *TaDOG1L4-YCE*, while no fluorescence was detected in the vector control (Fig. 5b). However, we did not observe YFP signal when co-expressing *TaPP2C-a10-YNE* and *TaDOG1L1-YCE*. Taken together, TaPP2C-a10 interacts with TaDOG1L4 in the nuclei.

### *TaPP2C-a10* exhibits the highest transcript level in wheat grains

**Fig. 6:**
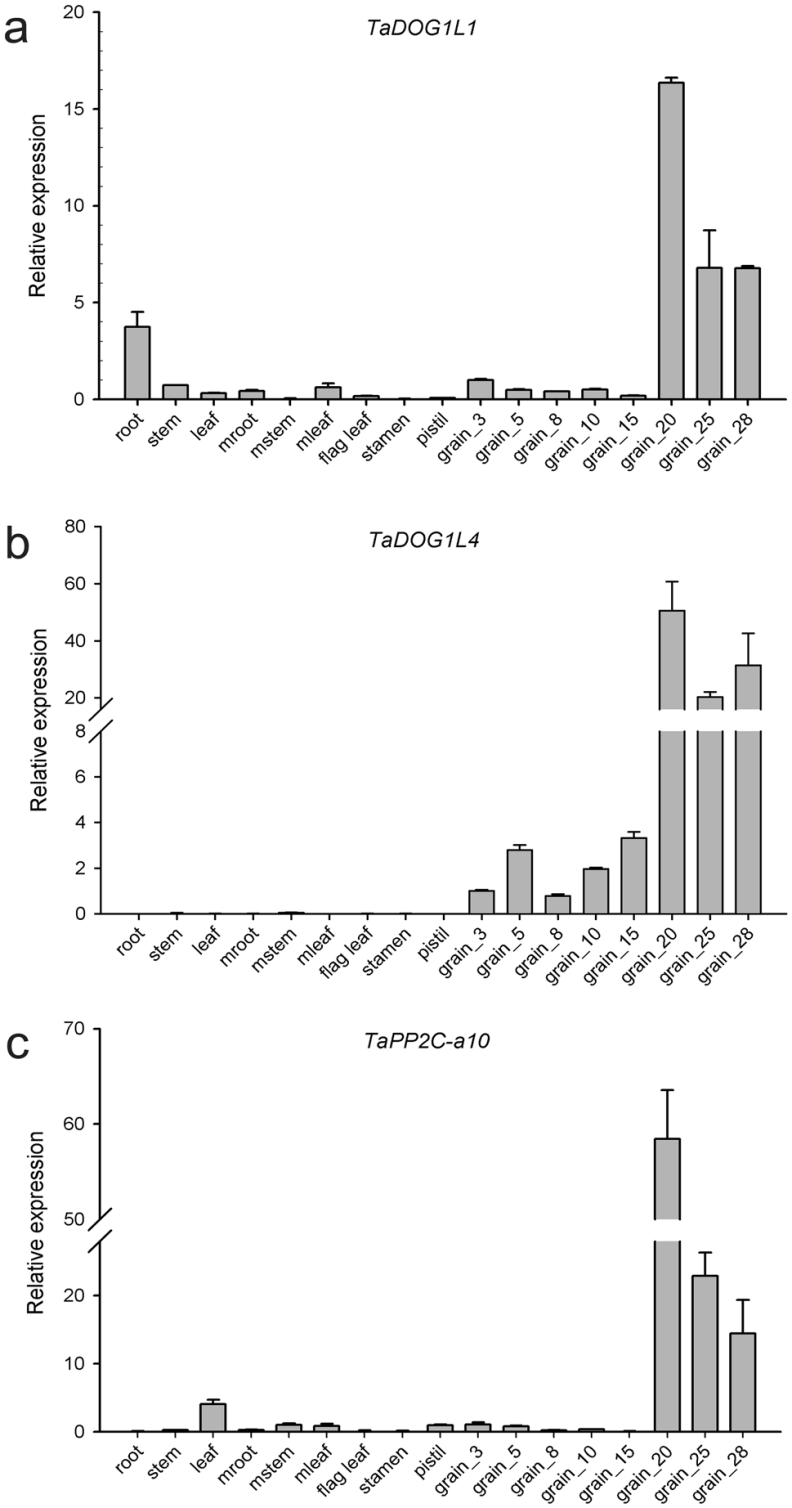
Quantitative RT-PCR analysis of *TaDOG1L1*, *TaDOG1L4* and *TaPP2C-a10* genes in various tissues at different developmental stages. Expressions of *TaDOG1L1* (**a**), *TaDOG1L4* (**b**) and *TaPP2C-a10* (**c**) in root, stem, leaf tissues from seedlings at three leaf stage, root, stem leaf, flag leaf, stamen and pistil tissues from mature plants at flowering stage and grains at different dpa stages were analyzed. Error bars represent the standard deviation of three independent replicates

The qRT-PCR analysis was applied to examine the expression patterns of *TaDOG1L1*, *TaDOG1L4* and *TaPP2C-a10* in different wheat tissues. *TaDOG1L1* mainly expressed in roots, stems and grains, particularly in 20 dpa grains (Fig. 6a). This finding partially corresponded to the RNA-seq data of *TaDOG1L1* which displayed higher transcript abundance in roots than stems and grains. *TaDOG1L4* specifically expressed in grains, and had significant increase (more than 20-fold) at late grain development stage (Fig. 6b). *TaPP2C-a10* expressed largely in grains and less in young leaves. The transcript level of *TaPP2C-a10* reached the highest (nearly 60-fold) at 20 dpa grains, and decreased with time after that (Fig. 6c). Although expression patterns of *TaDOG1L1*, *TaDOG1L4* and *TaPP2C-a10* were distinct from each other in these tissues, they crossed and overlapped in grains, especially at late development stage.

### TaPP2C-a10 promotes seed germination in Arabidopsis

Yeast two-hybrid assay showed that TaPP2C-a10 interacted with TaDOG1L1 and TaDOG1L4 which were involved in seed dormancy and germination (Ashikawa et al. 2010, 2014), and qRT-PCR analysis showed that *TaPP2C-a10* preferentially expressed in grains. These results indicated that TaPP2C-a10 might affect seed dormancy and germination by interacting with DOG1Ls. To investigate whether TaPP2C-a10 has such roles, we subsequently introduced *TaPP2C-a10* into Arabidopsis by *Agrobacterium* mediated floral-dip method for functional characterization. GUS staining was applied to detect the transgenic lines (Fig. S1a). Six independent transgenic Arabidopsis lines were obtained, and expression levels of *TaPP2C-a10* in these lines were verified by RT-PCR (Fig. S1b). Apart from to transgenic line 6, lines 1–5 all had relative high *TaPP2C-a10* expression levels. Therefore, homozygous lines 2, 3 and 4 (L2, L3 and L4) were randomly chosen to perform germination assay. Transgenic Arabidopsis of pSN1301 empty vector was used for negative control.

**Fig. 7:**
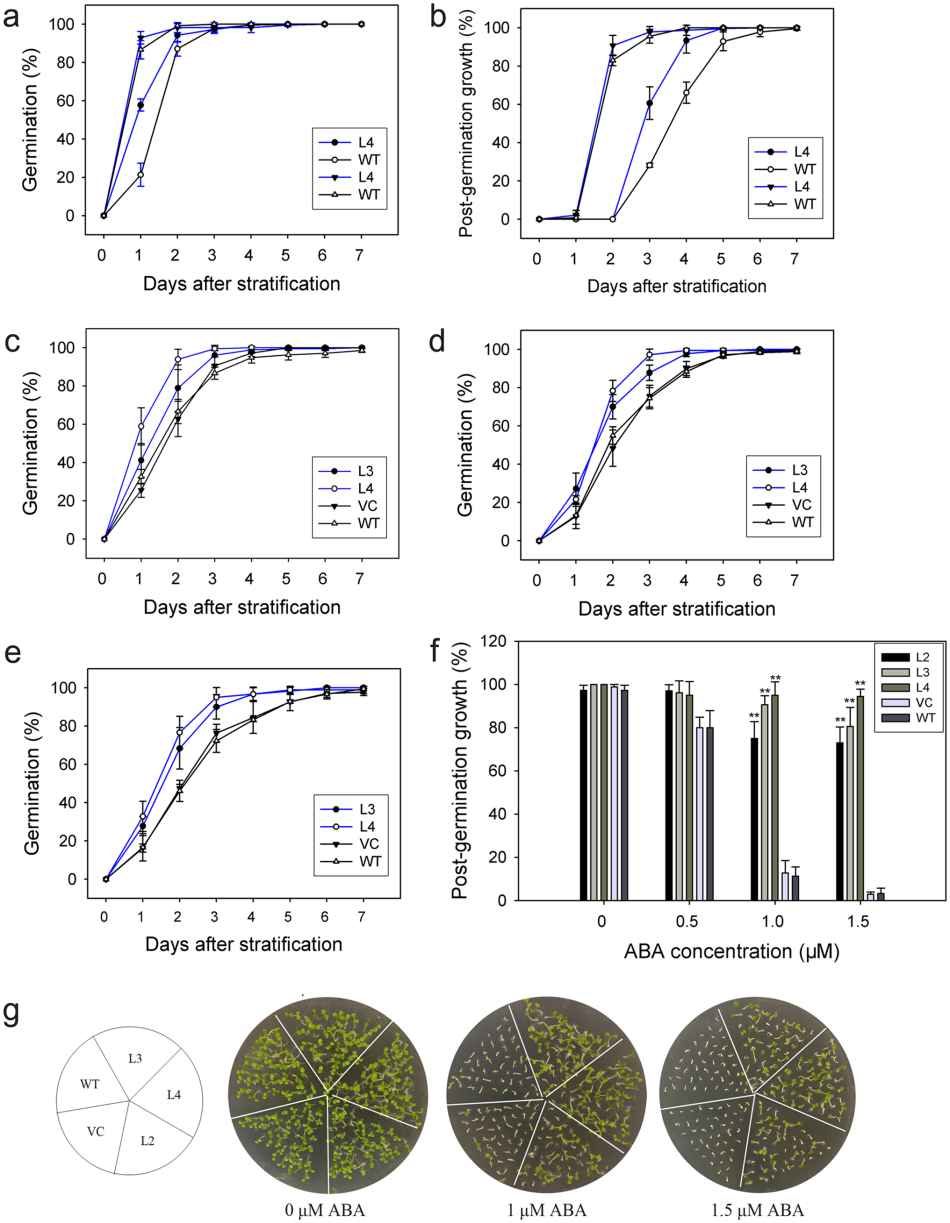
TaPP2C-a10 decreases ABA sensitivity of transgenic Arabidopsis during germination. Germination (**a**) and post-germination (**b**) efficiencies of *TaPP2C-a10* transgenic seeds (closed symbols) and wild-type (WT) seeds (open symbols) after stratification for 0 day (circles) or 4 days (triangles). Germination (**c**–**e**) and post-germination (**f**) efficiencies of seeds from *TaPP2C-a10* transgenic lines, vector control (VC) and WT plants in the presence of 0.5 (**c**), 1.0 (**d**), 1.5 (**e**) μM ABA after stratification for 4 days. Error bars indicate the standard deviation of three independent experiments (**a**–**f**). **g** Phenotypes of *TaPP2C-a10* transgenic lines, VC and WT plants. Seeds were grown on 1/2 MS plates containing various concentrations of ABA for 7 days

To analyze the effect of seed stratification on the germination of *TaPP2C-a10* transgenic Arabidopsis in the absence of exogenous ABA, germination and post-germination growth efficiencies after stratification for 0 and 4 days were calculated. Without stratification, *TaPP2C-a10* transgenic line L4 germinated and grew slightly faster than the wild type (Fig. 7a, b). No apparent difference was observed between L4 and the wild type after stratification treatment. As our previous study showed that *TaPP2C-a10* significantly responded to ABA stress, to elucidate the role of TaPP2C-a10 in the ABA response of transgenic Arabidopsis, germination and post-germination growth efficiencies under various concentrations of ABA after stratification were examined. Under low concentration of ABA treatment, no significant difference was found among *TaPP2C-a10* transgenic lines, VC and WT (Fig. 7c, g). However, at higher ABA content (1.0 and 1.5 μM) medium, the germination efficiencies of *TaPP2C-a10* transgenic lines were less inhibited by ABA comparing to VC and WT lines (Fig. 7d, e). Additionally, the post-germination growth efficiencies of *TaPP2C-a10* transgenic lines retained 70–90% with high ABA content, exhibiting significant difference (**p < 0.01) from VC and WT which had dramatically reduced post-germination growth efficiencies (less than 20% at 1.0 μM ABA) (Fig. 7f, g). These observations indicate that TaPP2C-a10 promotes seed germination in Arabidopsis and decreases sensitivity to ABA during germination.

### TaPP2C-a10 regulates seed germination and root growth though ABA signaling

**Fig. 8:**
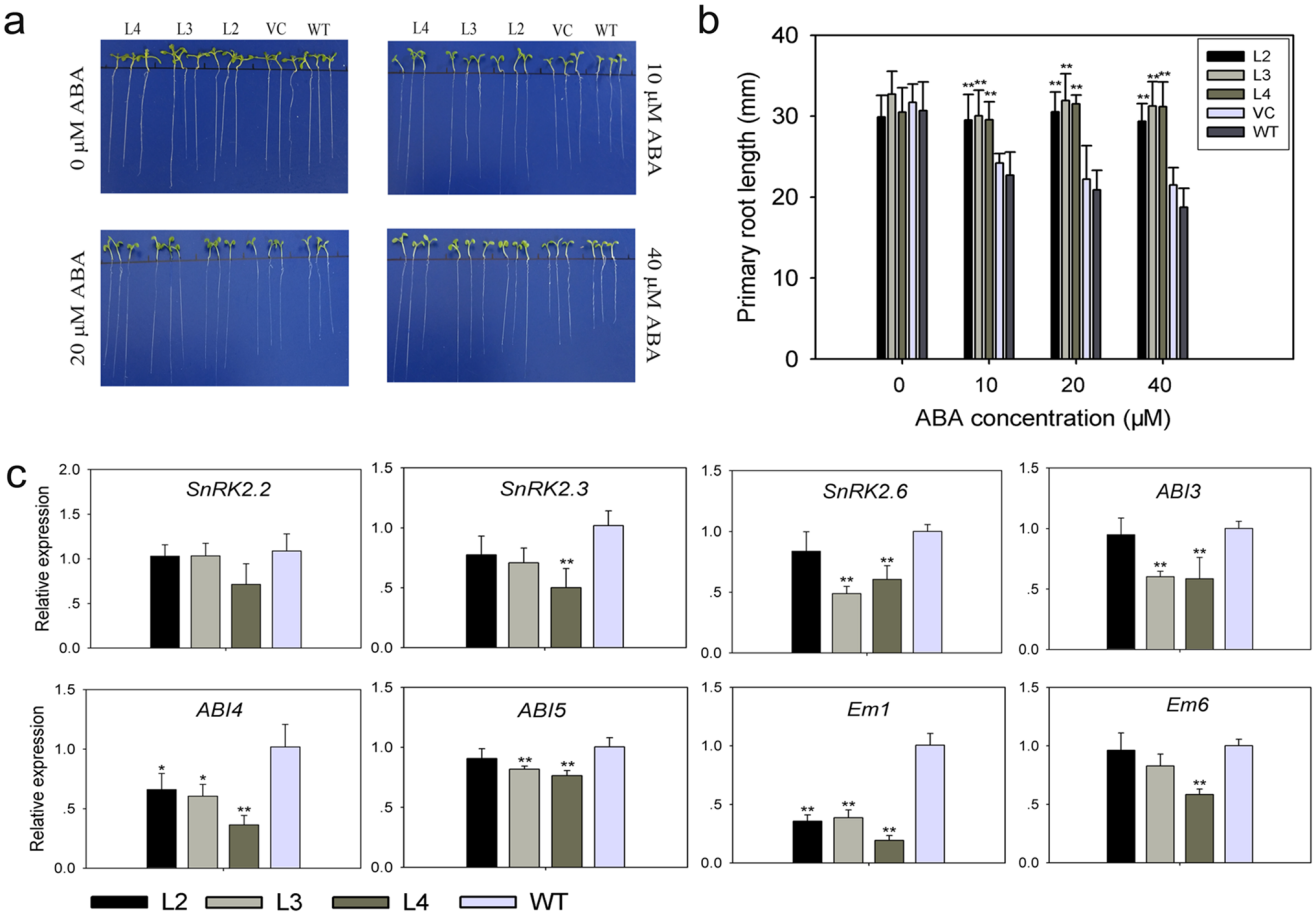
TaPP2C-a10 regulates seed germination and primary root growth though ABA signaling. **a** Primary root in *TaPP2C-a10* transgenic lines is insensitive to ABA. Five-day-old seedlings from hormone-free medium were transferred to 1/2 MS with various concentrations of ABA. Primary root length was measured after 7 days. **b** Statistical analysis of primary root lengths of *TaPP2C-a10* transgenic lines, VC and WT in **a**. **c** Expression analysis of ABA-responsive genes in *TaPP2C-a10* transgenic lines and WT. Whole plants of 7-day-old seedlings were used for analysis. Error bars indicate the standard deviation of three independent replicates (**b**, **c**). The asterisks indicate significant differences compared with the wild type (*P < 0.05, **P < 0.01; Tukey test)

Besides seed germination, ABA also affects plant growth including primary root growth (Hong et al. 2013). To validate whether constitutive expression of *TaPP2C-a10* gene in Arabidopsis could affect root growth, 5-day-old seedlings of same size and consistent growth status were transferred to medium containing various concentrations of ABA and the primary root lengths were measured after seven days. In the absence of ABA, primary root lengths of *TaPP2C-a10* transgenic lines were almost the same as the controls (Fig. 8a, b). With high concentrations of ABA (no less than 10 μM), primary root elongations of VC and WT were visibly inhibited by ABA, while those of *TaPP2C-a10* transgenic lines seemed to be unaffected, even under treatment with 40 μM ABA, suggesting that TaPP2C-a10 also decreases ABA sensitivity of root during plant development.

To further examine whether ABA signaling in *TaPP2C-a10* transgenic Arabidopsis was affected comparing to the wild type, transcript levels of ABA-responsive genes, such as *SnRK2*s (Nakashima et al. 2009), *ABI3* (Giraudat et al. 1992), *ABI4* (Finkelstein et al. 1998), *ABI5* (Finkelstein and Lynch 2000), *Em1* and *Em6* (Carles et al. 2002) were confirmed by qRT-PCR using 7-day-old seedlings (Fig. 8c). The result showed that the expression levels of all tested ABA-responsive genes were reduced at different degrees in *TaPP2C-a10* transgenic Arabidopsis. Additionally, the expression of *Em1* was significantly suppressed in all *TaPP2C-a10* transgenic lines (**P < 0.01). These findings agreed with the ABA-insensitive phenotypes of *TaPP2C-a10* transgenic lines. As these ABA-responsive genes were involved in regulating seed germination and plant growth, we could deduce that TaPP2C-a10 takes part in ABA signaling pathway to regulate seed germination and root growth.

### TaPP2C-a10 decreases drought tolerance in Arabidopsis

**Fig. 9:**
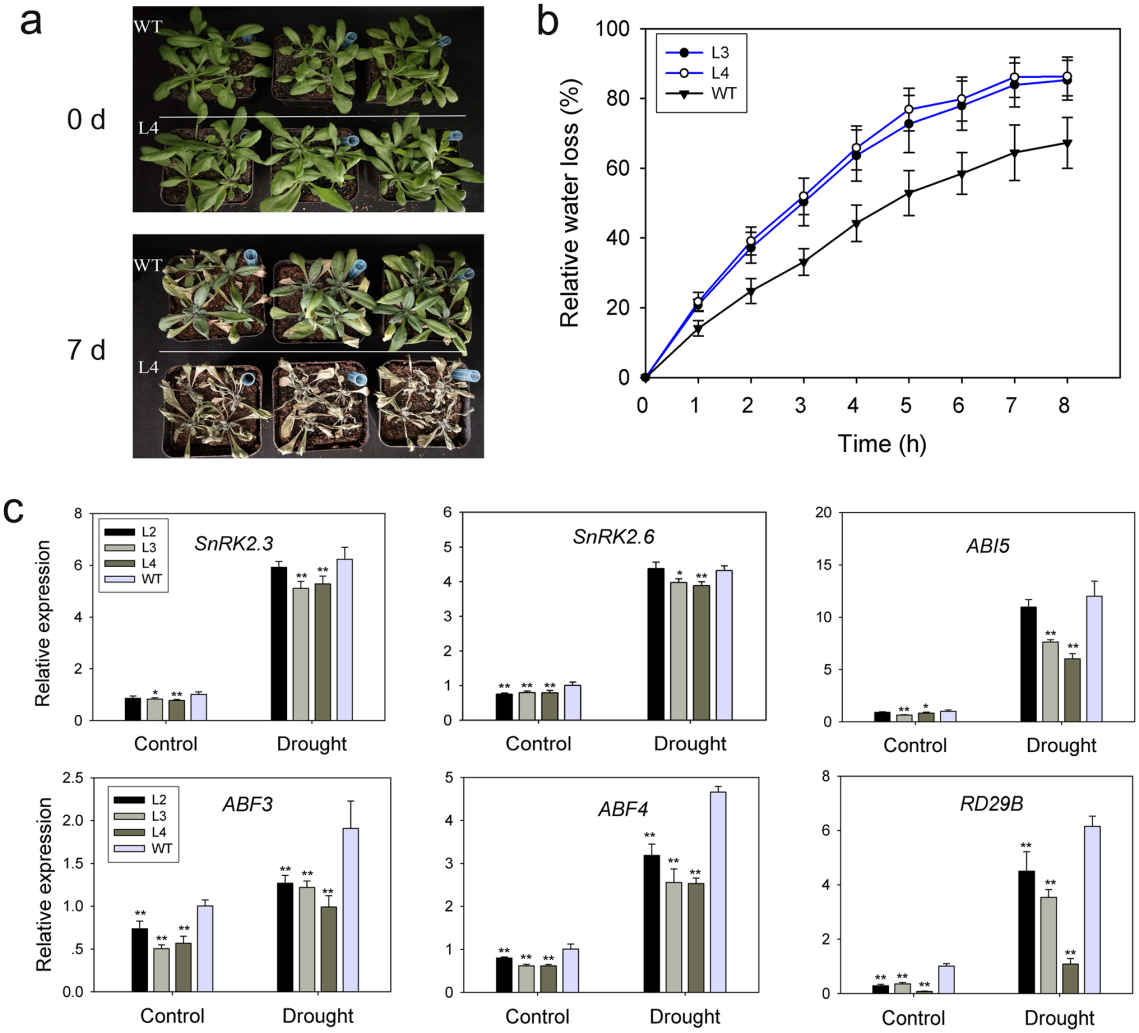
TaPP2C-a10 negatively regulates drought tolerance of transgenic Arabidopsis. **a** Phenotypes of *TaPP2C-a10* transgenic line and WT plants under drought conditions. Four-week-old plants grown on soil were withheld water for 7 days. **b** Relative water loss of detached rosette leaves from *TaPP2C-a10* transgenic lines (circles) and WT plants (triangles). Error bars indicate the standard deviation of three replicates. **c** Expression analysis of drought-responsive genes in *TaPP2C-a10* transgenic lines and WT plants. Ten-day-old plants with or without drought treatment were used for analysis. Error bars indicate the standard deviation of three independent replicates. The asterisks indicate significant differences compared with the wild type (*P < 0.05, **P < 0.01; Tukey test)

As the expression level of *TaPP2C-a10* was greatly increased after PEG treatment (Yu et al. 2019), the effect of drought stress on *TaPP2C-a10* transgenic Arabidopsis was checked. For drought treatment assay, adult plants grown in soil for 4–5 weeks were withheld water. After 7 days, *TaPP2C-a10* transgenic plant withered and died, while WT plants seemed much healthier (Fig. 9a). Furthermore, detached leaves of *TaPP2C-a10* transgenic plants exhibited higher relative water loss rate than WT (Fig. 9b). These results suggest that TaPP2C-a10 decreases drought tolerance ability of transgenic Arabidopsis.

To further confirm the role of TaPP2C-a10 in drought stress response, the transcript levels of drought-responsive genes in 10-day-old plants before and after drought treatment were analyzed by qRT-PCR. Under normal conditions, the expression levels of *SnRK2.2*, *SnRK2.6*, *ABI5*, *ABF3*, *ABF4* and *RD29B* in transgenic Arabidopsis were significantly reduced than those in WT (Fig. 9c). After drought treatment, this difference was still evident between *TaPP2C-a10* transgenic lines and WT, indicating that expression of *TaPP2C-a10* affects the drought response of transgenic Arabidopsis.

### *TaPP2C-a10* gene is conserved during evolutionary process

**Fig. 10:**
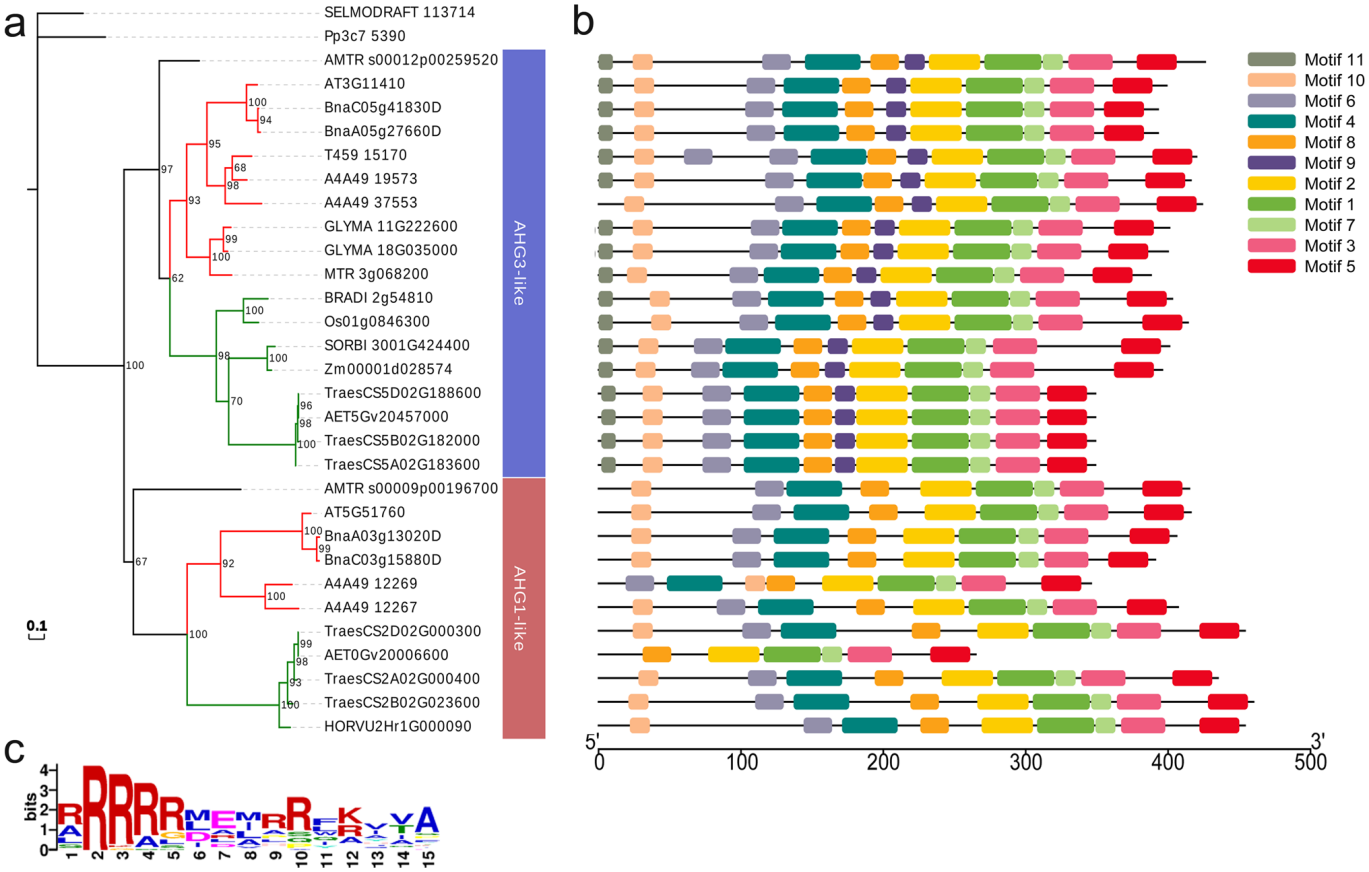
Phylogenetic analysis and motif distribution pattern of AHG-like proteins. **a** Phylogenetic analysis of AHG-like proteins. The phylogenetic tree was constructed using the ML method by IQ-TREE with 1000 bootstrap replicates. The red and green branches represent eudicotyledon and monocotyledon species, respectively. The scale bar indicates 0.1 amino acid substitutions per site. **b** Motif distribution pattern of AHG-like proteins in angiosperm. Eleven motifs were discovered by MEME searching tool. The scale bar below corresponds to the number of amino acid residues. **c** Sequence logo of motif 10 (color figure online)

Phylogenetic analysis revealed that TaPP2C-a10 belonged to AHG1 subfamily (Fig. 4), and TaPP2C-a10 functioned like AHG1 and AHG3 by regulating seed dormancy and germination with DOG1Ls. To further survey the evolutionary relationships between these AHG-like proteins, 31 AHG-like proteins including TaPP2C-a10 were identified by BLAST tool (Table 1). These proteins were from different families and genera in eudicotyledon, monocotyledon, amborellales, lycopodiopsida and bryopsida. The phylogenetic tree was constructed by ML method with 1000 bootstrap replicates (Fig. 10a). The AHG-like proteins in angiosperms happened to fall into two groups: AHG1- and AHG3-like groups. From evolution perspective, the evolution pattern of *AHG1*/*AHG3-like* gene accorded with the evolutionary process of angiosperms. This indicates that *AHG1*/*AHG3-like* gene including *TaPP2C-a10* is conserved during evolutionary process. Eleven conserved motifs were discovered within these AHG-like proteins (Fig. 10b, Table S3), and motifs 1–7 existed in the entire PP2C family. Apart from these seven motifs, motif 8 was only found in group A PP2Cs, motif 10 was specifically possessed by the AHG-like proteins, and the rest (motifs 9 and 11) merely existed in AHG3-like group. Moreover, motif 10 was rich in arginine which is a kind of alkaline amino acid (Fig. 10c).

**Table 1:** *AHG-like* genes in various plant species

Class	Species	Gene ID	Gene name
Eudicotyledon	*Arabidopsis thaliana*	*AT5G51760*	*AHG1*
		*AT3G11410*	*AHG3*
	*Brassica napus*	*BnaA03g13020D*	
		*BnaC03g15880D*	
		*BnaC05g41830D*	
		*BnaA05g27660D*	
	*Glycine max*	*GLYMA_18G035000*	
		*GLYMA_11G222600*	
	*Medicago truncatula*	*MTR_3g068200*	
	*Capsicum annuum*	*T459_15170*	
	*Nicotiana attenuata*	*A4A49_12267*	*AHG1_0*
		*A4A49_12269*	*AHG1_2*
		*A4A49_19573*	*PP2CA_1*
		*A4A49_37553*	*PP2CA_0*
Monocotyledon	*Brachypodium distachyon*	*BRADI_2g54810v3*	
	*Oryza sativa Japonica Group*	*Os09g0325700*	*PP2C1*
	*Aegilops tauschii*	*AET0Gv20006600*	
		*AET5Gv20457000*	
	*Hordeum vulgare *subsp.* vulgare*	*HORVU2Hr1G000090*	
	*Triticum aestivum*	*TraesCS2A02G000400*	*TaPP2C-a10*
		*TraesCS2B02G023600*	
		*TraesCS2D02G000300*	
		*TraesCS5A02G183600*	*TaPP2C-a8*
		*TraesCS5B02G182000*	
		*TraesCS5D02G188600*	
	*Sorghum bicolor*	*SORBI_3001G424400*	
	*Zea mays*	*Zm00001d028574*	*PP2C37*
Amborellales	*Amborella trichopoda*	*AMTR_s00012p00259520*	
		*AMTR_s00009p00196700*	
Lycopodiopsida	*Selaginella moellendorffii*	*SELMODRAFT_113714*	
Bryopsida	*Physcomitrella patens*	*Pp3c7_5390*

## Discussion

### Functional diversity of TaDOG1L family

Seed dormancy and germination are important traits for crops. *DOG1* is critical to the induction of seed dormancy. In the present study, six *TaDOG1L* genes (15 homoeologs) were identified by genome-wide searching (Fig. 1). Actually, another 39 proteins also contained the DOG1 domains, but technically belonged to the clade D bZIP transcript factors according to the description of *DOG1* family (Bentsink et al. 2006). Expression patterns of *TaDOG1L*s in different tissues showed almost all *TaDOG1L*s had expressions in grains. Further expression analyses of *TaDOG1L*s in different cell types of endosperms showed that most genes expressed in the aleurone layer. As aleurone cells are essential for grain germination (Pfeifer et al. 2014), *TaDOG1L*s potentially participate in seed germination. In fact, *TaDOG1L1* and *TaDOG1L4* have been verified to regulate seed dormancy and germination (Ashikawa et al. 2010, 2014). Expression analyses of *TaDOG1L*s also revealed that three genes including *TaDOG1L2* and *4* specifically expressed in grains, while others genes like *TaDOG1L1* had non-specific and broad expression in wheat except for *TaDOG1L-N1* which had no detectable value in all tissues (Fig. 2). The diverse expression patterns of *TaDOG1L* genes suggest functional difference between them.

Additionally, TaDOG1L1, TaDOG1L2 and TaDOG1L4 displayed different interactions with group A TaPP2Cs. While TaDOG1L1 interacted with TaPP2C-a5, -a9 and -a10, TaDOG1L2 had no interactions with any TaPP2Cs. Meanwhile, TaDOG1L4 only interacted with TaPP2C-a10 (Fig. 3). Transient expression of TaDOG1Ls fused with GFP showed that TaDOG1L1 was observed in cytoplasm and nuclei, whereas TaDOG1L4 was only detected in nuclei (Fig. 4). These results also suggest the functional diversity of TaDOG1L family. In Arabidopsis, unlike AtDOG1 mutant, AtDOGL1, AtDOGL2, and AtDOGL3 mutants germinated normally (Bentsink et al. 2006). Therefore, DOG1L family members play roles in more than regulating seed dormancy and germination. Two studies have demonstrated that DOG1L family is involved in regulating flowering time (Huo et al. 2016) and drought tolerance (Zhang et al. 2019). Besides, two-hybrid assay showed that TaDOG1L1 had interactions with TaPP2C-a5, -a9 and -a10 which were all from the AHG1 subfamily, but not ABI1 subfamily (Fig. 4); in other words, TaDOG1L1 only interacted with TaPP2Cs from AHG1 subfamily. This is partly in accordance with the result in Arabidopsis that AtDOG1 had interactions with all AHG1 subfamily members rather than ABI1 subfamily members from group A PP2Cs (Nishimura et al. 2018). However, there is apparent difference that TaDOG1L1 could not interact with all AHG1 subfamily members, suggesting different interaction patterns of DOG1 and group A PP2Cs in wheat and Arabidopsis. Additionally, to confirm the relationship between TaDOG1Ls and TaPP2C-a10, other protein–protein interaction methods need to be considered in the further study.

### TaPP2C-a10 can regulate seed germination and plant growth though ABA signaling

Two-hybrid and BiFC assays exhibited that TaPP2C-a10 could interact with TaDOG1L4 (Figs. 3, 5b). Phylogenetic analysis revealed that TaPP2C-a10 was highly homologous to Arabidopsis AHG1 (Fig. 4), which interacts with N-terminus of AtDOG1 to control seed dormancy and germination (Née et al. 2017; Nishimura et al. 2018). Meanwhile, qRT-PCR analysis displayed that *TaPP2C-a10* abundantly expressed in grains (Fig. 6c). Thus, TaPP2C-a10 might function in seed dormancy and germination. Further investigation showed that *TaPP2C-a10* transgenic Arabidopsis germinated faster than WT, especially in the presence of high content ABA (Fig. 7). The primary roots of transgenic seedlings also elongated longer than those of the controls on the mediums with various concentrations of ABA (Fig. 8a, b). Therefore, TaPP2C-a10 indeed plays roles in seed germination and plant growth.

Expression analysis displayed that transcript levels of some ABA-responsive genes were dramatically decreased in *TaPP2C-a10* transgenic Arabidopsis (Fig. 8c); while the *AtDOG1* expression level in freshly harvested seeds of *TaPP2C-a10* transgenic Arabidopsis had no significant difference from those of WT plants (Fig. S2). These ABA-responsive genes included subclass III *SnRK2*s (*SnRk2.2*, *SnRk2.3* and *SnRk2.6*), *ABI3*, *ABI4*, *ABI5*, *Em1* and *Em6* (Nakashima and Yamaguchi-Shinozaki 2013). Previous study showed that DOG1 genetically interacted with ABI3, and affected the expression of downstream *ABI5* (Dekkers et al. 2016). Another study confirmed synergistic action between ABI4 and ABI5 in regulating gene expression (Reeves et al. 2011). As ABI3, ABI4 and ABI5 play essential roles in seed development, and their mutants decreased sensitivity to ABA inhibition of germination (Giraudat et al. 1992; Finkelstein et al. 1998; Finkelstein and Lynch 2000), we deduce that the accumulation of TaPP2C-a10 protein in transgenic Arabidopsis suppresses the DOG1-ABI3-ABI5 pathway, thus decreasing sensitivity to ABA during germination. Moreover, ABI5 directly regulated expressions of *Em1* and *Em6* by binding to their promoters (Bensmihen et al. 2002; Carles et al. 2002), this can explain the lower expression levels of *Em1* and *Em6* in *TaPP2C-a10* transgenic lines. On the other hand, subclass III SnRK2s are positive regulators of ABA signaling pathway, which phosphorylate ABFs and ABI5 to activate ABRE-driven gene expression (Umezawa et al. 2010; Fujita et al. 2013). Group A PP2Cs can dephosphorylate and inactivate subclass III SnRK2s. Additionally, SnRk2.2, SnRk2.3 and SnRk2.6 were shown to control seed dormancy and development through ABA signaling (Fujii et al. 2007; Nakashima et al. 2009). Thus, TaPP2C-a10 protein might also regulate seed germination by affecting SnRK2-ABI5 pathway. These findings suggest the cross-talk and signal integration between DOG1 and ABA signaling pathways, furthermore, group A PP2Cs, such as TaPP2C-a10 and AHG1, act like cross points therein.

Interestingly, analysis of upstream regulatory sequences of *TaDOG1L1* and *TaDOG1L4* revealed that there were some ABRE-motifs existing in their promoters (Table S4). Previous study reported that *DOG1* antisense strongly responded to ABA stress (Yatusevich et al. 2017). We further analyzed the response of *TaDO1L1* to ABA stress, and the expression of *TaDO1L1* was significantly decreased after ABA treatment (Fig. S3). To figure out whether TaDOG1Ls have roles in ABA signaling, deep researches are required. Additionally, our previous study showed that *TaPP2C-a10* was significantly up-regulated after GA treatment, and further investigation on the promoter of *TaPP2C-a10* revealed the presence of GA-responsive element (Yu et al. 2019). A previous report has revealed that a group A PP2Cs was involved in both ABA and GA signaling pathways (Kim et al. 2013). Therefore, TaPP2C-a10 might also participate in GA signaling pathway to regulate seed dormancy and germination, still this assumption needs experimental verification. Nevertheless, the reason why expression levels of subclass III *SnRK2*s were reduced in *TaPP2C-a10* transgenic lines remains unclear, as little is known about regulatory mechanism of *SnRK2*s at expression level.

### *TaPP2C-a10* is also involved in drought stress response

Group A PP2Cs were shown to repress the intrinsic desiccation tolerance of the vegetative tissue (Komatsu et al. 2013). *TaPP2C-a10* transgenic plants exhibited weaker tolerance to drought stress comparing to WT, and its detached leaves had relative higher water loss rate (Fig. 9a, b). Meanwhile, *TaPP2C-a10* transcript level was notably induced by PEG treatment (Yu et al. 2019). Therefore, TaPP2C-a10 negatively regulates drought stress response. A recent study also showed that group A *PP2C* genes *ZmPP2C-A2* and *ZmPP2C-A6* negatively regulated drought responses in maize (*Zea mays*) (He et al. 2019). Our further analysis showed that expression levels of several drought stress-responsive genes were lower in *TaPP2C-a10* transgenic lines than the controls under normal condition, and were still lower than WT after drought treatment (Fig. 9c). Previous studies showed that subclass III *SnRK2*s responded to multiple stresses including drought stress in both ABA-independent and -dependent pathways (Fujita et al. 2009; Nakashima et al. 2009). SnRK2 kinases can activate the ABF transcription factors to regulate gene expression in ABA-dependent manner, thus responding to osmotic stress (Fujita et al. 2013; Yoshida et al. 2014). The reduced expression levels of *SnRk2.2*, *SnRK2.6*, *ABF3*, *ABF4* and *ABI5* suggest that the SnRK2-ABF pathway was suppressed in *TaPP2C-a10* transgenic Arabidopsis. Additionally, TaPP2C-a10 was found to interact with subclass III SnRK2s (Yu et al. 2019). Consequently, TaPP2C-a10 probably suppresses SnRK2-ABF pathway to affect plant drought stress response. Besides, the DOG1L family was shown to be involved in drought stress tolerance, even though the molecular mechanism was unclear. NtabDOG1L positively regulated drought stress tolerance in *N. tabacum* (Zhang et al. 2019). Inactivation of *DOG1* by its antisense transcript (*asDOG1*) resulted in enhanced drought sensitivity (Yatusevich et al. 2017). Thus, there is a possibility that TaPP2C-a10 regulates DOG1Ls to respond to drought stress.

### *AHG-like* gene is conserved in angiosperm

The evolutionary process of *AHG-like* gene in angiosperm suggests conserved structures and functions of AHG1/AHG3-like proteins (Fig. 10a). This was verified by the functional study of *TaPP2C-a10*. Moreover, the motif distribution pattern supports their evolutionary relationship (Fig. 10b). Motif 8 identified in Fig. 10 is similar to motif 7 identified in Fig. 4, this confirms their group A-specific characteristic. Besides, motif 10, which is arginine-rich, is peculiar to AHG-like protein and located at the N-terminus of peptide chains (Fig. 10c), suggesting that motif 10 might be required for functions of AHG-like proteins.

In conclusion, our study revealed functional diversity of the DOG1L proteins in wheat and physical interactions between TaDOG1L4 and TaPP2C-a10. Further investigation showed that TaPP2C-a10 regulated seed germination and plant growth though ABA signaling. Additionally, TaPP2C-a10 also negatively regulated drought stress response. The phylogenetic analysis of AHG-like proteins suggests that *TaPP2C-a10* gene is conserved during evolutionary process. These results provide valuable information for the functional studies of TaDOG1Ls and AHG-like proteins, and additional insights into the roles of group A PP2Cs.

## Electronic supplementary material

Below is the link to the electronic supplementary material.

Supplementary file1 (TIF 1425 kb)

Supplementary file2 (tif 167 kb)

Supplementary file3 (tif 199 kb)

Supplementary file4 (XLSX 11 kb)

Supplementary file5 (XLSX 11 kb)

Supplementary file6 (XLSX 11 kb)

Supplementary file7 (XLSX 12 kb)
