# Supplementary figures and images for: Wheat PP2C-a10 regulates seed germination and drought tolerance in transgenic Arabidopsis
Source: Plant Cell Rep. 2020 Feb 17;39(5):635–51. doi: 10.1007/s00299-020-02520-4 (PMC7165162; doi:10.1007/s00299-020-02520-4)

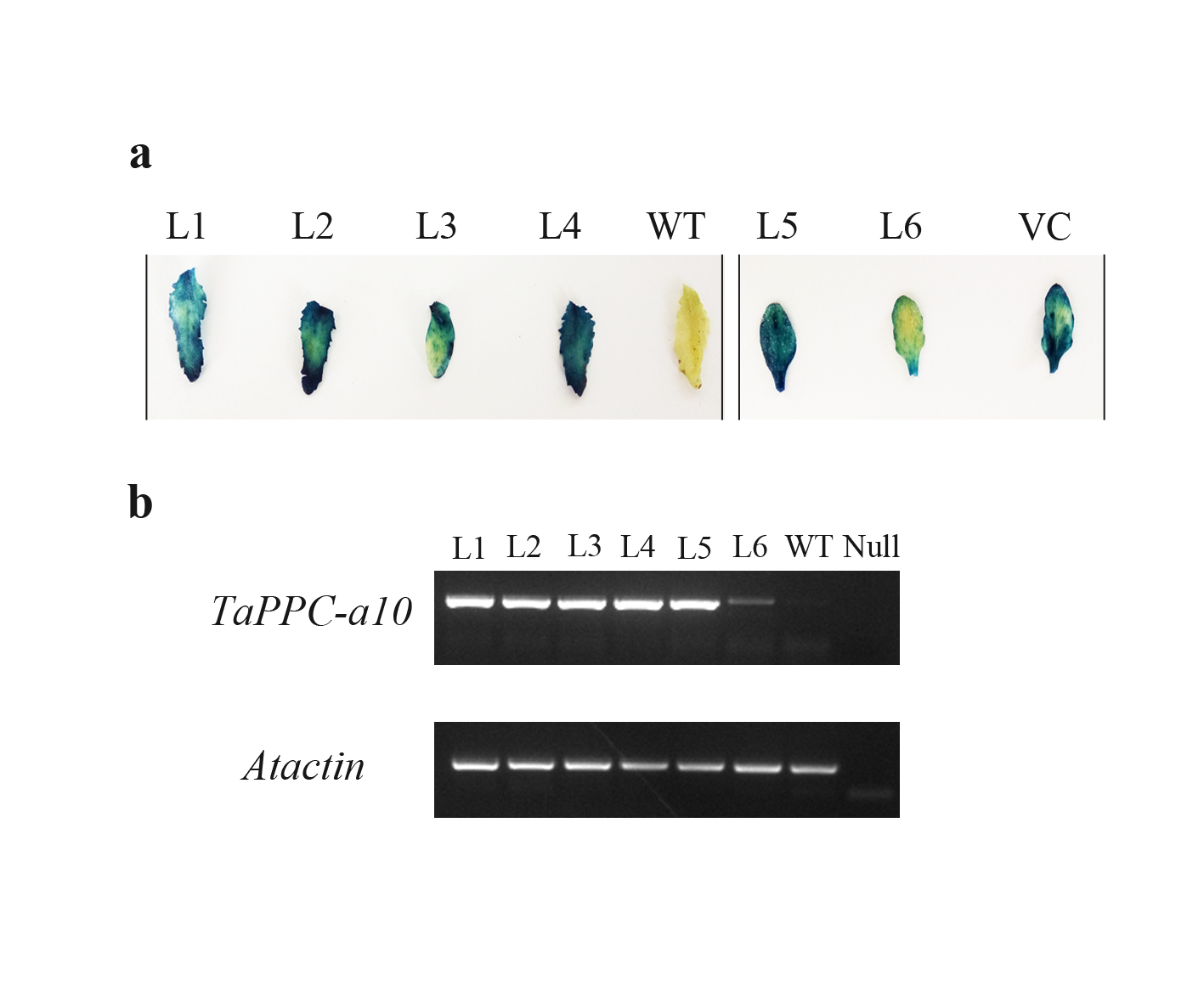

Supplement: Supplementary file 1 — Supplementary file1 (TIF 1425 kb) [file 299_2020_2520_MOESM1_ESM.tif]
